# Risk factors for epiretinal membrane formation and peeling following pars plana vitrectomy for primary rhegmatogenous retinal detachment, an OCT guided analysis

**DOI:** 10.1186/s40942-022-00418-9

**Published:** 2022-09-30

**Authors:** Andrei-Alexandru Szigiato, Fares Antaki, Simon Javidi, Samir Touma, Renaud Duval, Ghassan Cordahi, Sebastien Olivier, Flavio A. Rezende

**Affiliations:** Department of Ophthalmology, Hôpital Maisonneuve Rosemont, Montreal, Québec Canada

**Keywords:** Epiretinal membrane, Retinal detachment, Vitrectomy, Optical coherence tomography, OCT, Retina, Pneumatic retinopexy, Cryotherapy

## Abstract

**Background:**

To evaluate the rate and risk factors of epiretinal membrane (ERM) formation and need for ERM peeling after pars plana vitrectomy (PPV) for uncomplicated primary rhegmatogenous retinal detachment (RRD).

**Methods:**

Retrospective, single-center, cohort study of 119 consecutive patients (119 eyes) that underwent RRD repair using PPV. The primary outcomes were ERM formation, classified using an optical coherence tomography grading system, and the rate of ERM peeling. Visual acuity, postoperative complications, and risk factors for ERM formation and peeling were also identified.

**Results:**

Postoperative ERM formation occurred in 69 eyes (58.0%); 56 (47.1%) were stage 1, 9 (7.6%) stage 2, 3 (2.5%) stage 3, and 1 (0.8%) stage 4. Only 6 (5.0%) eyes required secondary PPV for a visually significant ERM, with a mean time to reoperation of 488 ± 351 days. Risk factors for ERM formation included intraoperative cryotherapy, more than 1000 laser shots, 360° laser photocoagulation, and choroidal detachment (p < 0.01). Eyes with more than 3 tears had a trend towards increased ERM surgery (p = 0.10).

**Conclusions:**

Visually significant ERM formation following PPV for primary RRD was uncommon in this cohort (5%). Half of the ERMs were detected after the first post-operative year, indicating that this complication may be underreported in studies with only 1-year follow-up.

## Introduction

Pars plana vitrectomy (PPV) is currently the most commonly used technique to repair a rhegmatogenous retinal detachment (RRD), with a high rate of anatomic and visual success [1]. Epiretinal membrane formation (ERM) is a common sequelae of RRD repair using PPV, with a large variability of incidence in the literature [2–5]. This is in part due to the methods used to diagnose ERM, with rates of detection being lower with biomicroscopic fundus examination (6–13%) [4, 6] compared to optical coherence tomography (OCT) (35.1–70.3%). [7, 8]

ERM formation is thought to be due to the proliferation of liberated retinal pigment epithelial (RPE) cells following the formation of a retinal break, which migrate towards the macular surface using the internal limiting membrane (ILM) as a scaffold [2, 9]. This is one of the most frequent causes of vision loss following successful RRD repair and ERM removal might be required in approximately 16% of cases [10]. Currently, PPV and ERM removal combined with ILM peeling is considered the mainstay treatment for ERMs [11].

Given this potential complication of primary vitrectomy, which may necessitate a second surgical intervention, some authors have advocated for prophylactic ILM peeling during primary PPV for RRD to reduce the incidence of ERM formation following successful RRD repair [2]. While evidence shows that it greatly reduces the incidence of ERM, there is limited evidence on the visual acuity (VA) gains and iatrogenic changes with prophylactic ILM peeling [2]. Furthermore, while there are several studies that examined the rate of ERM formation, there are few to none that have directly analyzed the preoperative or intraoperative risk factors for clinically significant ERM requiring peeling following primary PPV for uncomplicated RRD. Thus, the goal of this study was to characterize the rate and type of ERM formed using a standardized OCT classification [12], and to analyze the preoperative/intraoperative risk factors for ERM formation requiring surgical removal following PPV for RRD.

## Methods

This is a retrospective, single center review of consecutive patients who underwent 25-gauge PPV for primary RRD from Dec 2012 to Aug 2015 by 4 experienced surgeons (FAR, RD, GC, SO). The study was approved by the Institutional Review Board of the Maisonneuve Rosemont Hospital, conforming to the principles of the Declaration of Helsinki.

All patients underwent 3-port 25-gauge PPV with core vitrectomy, induction/confirmation of posterior hyaloid detachment and peripheral vitreous base shaving with scleral indentation. Gas endotamponade with 15% octafluoropropane (C_3_F_8_) was used in all cases. Endolaser laser retinopexy was performed in all cases to surround retinal breaks, retinal holes and areas of lattice degeneration. Intraoperative cryotherapy, the use of perfluorocarbon, and 360° laser was used at the surgeon’s discretion. When performed, 360° laser consisted of placing 3 rows of medium-white burns anterior to the level of the vortex veins. No ILM peeling was performed in any of the cases concomitantly with the primary RRD repair. Postoperative positioning was determined for each patient depending on the location of the retinal break and detachment. Eyes that developed secondary ERMs following successful RRD repair with clinically significant visual symptoms of decreased central vision or metamorphopsia underwent a second PPV with ERM and ILM peeling. ILMs were stained with infracyanine green (ICG) under BSS or air tamponade. The ILM was peeled up to the vascular arcades with a 25-gauge ILM forceps (Grieshaber Maxgrip Forceps, Alcon, Geneva, Switzerland). All patients were placed on cyclopentolate 1% twice daily for a week, moxifloxacin 0.5% four times daily for a week and prednisolone acetate 1% four times daily for 1 week, tapered by one drop a week for a month. Some surgeons also added nepafenac 0.1% three times a day for 1 month. Postoperative visits were at 1 day, 1 week, 1 month, 4 months, and then every year afterward. Macular OCT was typically performed 3–6 months post-operatively, then every 6 months at the surgeon’s discretion. Overall follow-up duration and complications were counted from the date of the first surgery. Patient charts were reviewed up to 5 years after their initial retinal detachment repair.

### Data collection

Charts were reviewed by 4 independent reviewers and de-identified data was collected, including age, sex, pre- and post-operative Snellen visual acuity (VA), preoperative examination findings, intraoperative notes, and post-operative complications/reoperations, with follow-up for as long as was available in each chart. Patients were censored from the analysis following repeated retinal surgery.

Retinal detachments were classified by extent of detachment (number of quadrants), number and location of tears, macular status (on or off), presence of giant retinal tear, presence of vitreous hemorrhage and choroidal detachment. Time from detachment to surgical repair was recorded, defined as date of initial confirmation of retinal detachment by ophthalmic exam to the date of surgery. History of prior pneumatic retinopexy and number of attempts prior to vitrectomy were also included.

Patients were excluded if they had less than 6 months of follow-up, a history of trauma, prior retinal surgery, previous retinal detachment, tractional retinal detachment, combined scleral buckling, suprachoroidal buckling, use of silicone oil, presence of ERM or macular hole preoperatively, no OCT post-operatively to grade ERM prior to peeling, grade C proliferative vitreoretinopathy, re-detachment within the first 6 postoperative months, and post-operative vitrectomy for retained perfluorocarbon. If a patient had a retinal detachment in the fellow eye during the study period, only the first eye was included.

**Fig. 1:**
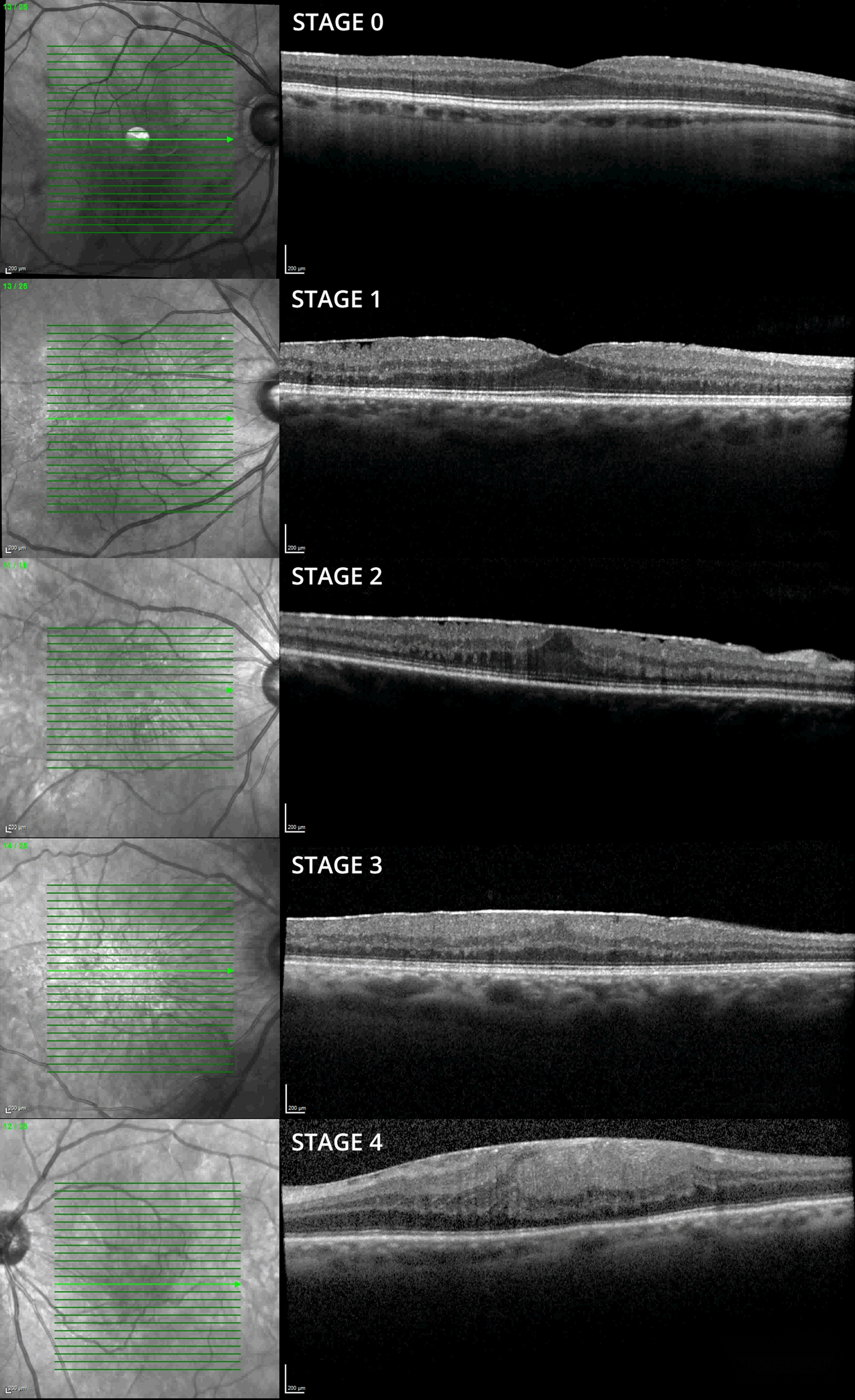
OCT based classification of ERMs in this cohort, as proposed by Govetto et al. Stage 1 ERM with hyporeflective intraretinal cystoid spaces (ICS). The foveal pit is present and the retinal layers are well-defined. Stage 2 ERM with hyporeflective ICS. The foveal pit is absent but the retinal layers remain well-defined. Stage 3 ERM demonstrating an absent foveal pit, well-defined retinal layers and ectopic inner foveal layers. Those layers represent hyperreflective bands extending from the inner nuclear layer (INL) and inner plexiform layer (IPL) across the foveal region. Stage 4 ERM showing an absent foveal pit, disrupted retinal layers and ectopic inner foveal layers. Stage 0 is a normal retina with no ERM

Macular spectral-domain OCT (SD-OCT) images were analyzed by 2 reviewers collaboratively using the software provided by the CIRRUS HD-OCT 500 (Carl Zeiss AG, Jena, Germany) and Spectralis Heidelberg OCT (Heidelberg, Germany) machines. An OCT based classification system proposed by Govetto et al. was used to identify the type of ERM [12]. Briefly, stage 1 ERMs were defined by a preserved foveal pit and clearly demarcated retinal layers; stage 2 absence of foveal pit and well defined retinal layers; stage 3 absence of foveal pit and presence of ectopic inner foveal layers with otherwise well-defined retinal layers; stage 4 disrupted retinal layers (Fig. 1). Reference OCT images were collected at 1 year for patients with no ERM seen on OCT (stage 0) during their follow-up.

The primary outcome was reoperation for ERM peeling. Patients with reoperation for re-detachment were censored from the survival analysis. Preoperative risk factors for ERM surgery were then assessed, including age, gender, macular status, number of tears, time to operation, preoperative pneumatic retinopexy, number of laser shots, and other intraoperative procedures.

Secondary outcomes included the incidence and type of ERM formed, based on OCT. Timing of ERM formation, VA, and complications, including re-detachment were also assessed. Proliferative vitreoretinopathy (PVR) was graded using the updated Retina Society classification [13].

### Statistical analysis

Baseline demographics were assessed with descriptive statistical analysis. Conversion from Snellen to LogMAR VA was performed using previously established formulas [14]. Visual acuities of count fingers (CF), hand motion (HM), light perception (LP), and no light perception (NLP) were approximated as follows: CF = 20/1450, HM = 20/3800, LP = 20/40000, NLP = 20/60000 [14, 15]. One way ANOVA with Bonferroni post hoc analysis was used to assess differences in VA and other factors between different ERM types. Kaplan–Meier survival analyses were performed to assess the rate of reoperation for ERM and for re-detachment. A univariate Cox regression analysis was employed to determine pre-operative risk factors and hazard ratios for ERM formation and ERM peel. Statistical analysis was conducted using SPSS (IBM version 22).

## Results

We included 119 eyes of 119 patients who underwent PPV for primary RRD. The mean ± standard deviation follow-up for all eyes included for analysis was 1257.8 ± 677 days.

Baseline characteristics of study eyes are summarized in Table 1. Mean age of patients was 61.1 years and 69% of patients were male. Approximately half of patients were phakic (n = 52, 44%), and one quarter had a failed pneumatic prior to vitrectomy (n = 38, 27%). The mean delay between symptom onset/confirmation of RRD to surgery was 8 ± 9 days. The majority of detachments were macula off (n = 78, 66%) and were 2 or more quadrants (n = 101, 85%).

**Table 1:** Baseline patient characteristics

Characteristic	Total (n = 119)	
Mean follow-up, days (SD)	1257.8	677.3
Mean age, years (SD)	61.1	10.4
Male, n (%)	82	68.9%
Right Eye, n (%)	58	48.7%
Lens status, n (%)		
Phakic	52	43.7%
Pseudophakic	64	53.8%
Aphakic	2	1.7%
Anterior chamber lens	1	0.8%
Failed Pneumatic, n (%)	38	31.9%
Number of failed pneumatics, n (%)		
One	32	26.9%
Two	6	5.0%
Days from detachment to surgery, mean (SD)	7.8	9.0
Q1	2	
Q2	5	
Q3	11	
Q4	37	
RRD quadrants, n (%)		
One	18	15.1%
Two	60	50.4%
Three	22	18.5%
Four	16	13.4%
Macula off, n (%)	78	65.5%
Giant retinal tear, n (%)	9	7.6%
Vitreous hemorrhage, n (%)	11	9.2%
Choroidal detachment, n (%)	2	1.7%
PVR grade A or B, n (%)	2	1.7%

*SD* standard deviation, *Q* quartile, *PVR* proliferative vitreoretinopathy

Intraoperative characteristics are summarized in Table 2. The majority of patients had 1 identified tear (n = 40, 34%), with the majority of tears occurring in the superotemporal quadrant (n = 65, 55%). The eyes that had 360° laser had 1079.6 ± 470 mean laser shots (± SD, n = 84). The mean number of laser shots per all cases was 969.8 ± 519. There were 14 eyes (12%) with intraoperative cryotherapy.

**Table 2:** Intraoperative patient characteristics, failure to cure, and postoperative complications following pars-plana vitrectomy for rhegmatogenous retinal detachment

Characteristic	Total (n = 119)	
Combined cataract surgery, n (%)	20	16.8%
Duration of surgery (mins), mean (SD)	71.9	23.9
Number of tears, mean (SD)	2.6	1.8
One, n (%)	40	33.6%
Two, n (%)	29	24.4%
Three, n (%)	13	10.9%
Four or more, n (%)	32	26.8%
Location of tear, n (%)		
Supero-temporal	65	54.6%
Supero-nasal	38	31.9%
Infero-nasal	30	25.2%
Infero-temporal	40	33.6%
Number of laser shots, mean (SD)	969.8	519.0
Q1	550	
Q2	843	
Q3	1323	
Q4	1776	
Intraoperative 360° Photocoagulation, n (%)	84	70.6%
Intraoperative ILM peeling, n (%)	0	0.0%
Intraoperative cryotherapy, n (%)	14	11.8%

Failure to cure and postoperative complications		
ERM formation, n (%)	69	58.0%
Stage 1	56	47.1%
Stage 2	9	7.6%
Stage 3	3	2.5%
Stage 4	1	0.8%
Reoperation for ERM	6	5.0%
Mean time to reoperation for ERM, days (SD)	488	351
PVR Grade C, n (%)	2	1.7%
Re-detachment, n (%)	2	1.7%
Macular hole, n (%)	4	3.4%
Full thickness, n (%)	2	1.7%
Lamellar, n (%)	2	1.7%
Severe macular edema ^a *^, n (%)	1	0.8%
Subretinal perflurocarbon, n (%)	1	0.8%

*SD* standard deviation, *Q* quartile, *ILM* internal limiting membrane, *ERM* epiretinal membrane, *PVR* proliferative vitreoretinopathy

^a *^Severe macular edema defined as central foveal thickness greater than 400um with no ERM or tractional component present

ERM classification and OCT characteristics are summarized in Table 3. Overall, there were 69 patients with confirmed ERM by OCT (58%); the majority were stage 1 (n = 56, 47%), followed by stage 2 (n = 9, 8%). There were 3 patients with stage 3 (3%) and 1 patient with stage 4 ERM (0.8%). Central foveal thickness and outer nuclear layer thickness was increased in stage 2, 3 and 4 ERMs compared to stage 0 and 1 (p < 0.01). Ectopic inner foveal layer thickness increased in stage 3 and 4 ERMs compared to stage 0 (p < 0.01). The VA of patients with stage 3 and stage 4 ERM was significantly decreased compared to all other groups (grouped mean LogMAR VA 0.8 ± 0.7 (Snellen 20/125), vs 0.2 ± 0.3 (20/30) p < 0.04).

**Table 3:** Epiretinal membrane prevalence, severity and retinal characteristics using optical coherence tomography

	Stage 0		Stage 1		Stage 2		Stage 3		Stage 4	
n (% total with OCT)	50	(42.0%)	56	(47.1%)	9	(7.6%)	3	(2.5%)	1	(0.8%)
Timing of OCT/ERM post surgery (days), mean (SD)	439.4	(368.1)	392.5	(199.1)	263.7	(184.8)	459.7	(692.0)	207.0	(NA)
Central foveal thickness (um), mean (SD)	245.9	(46.1)	258.3	(44.5)	345.6^+^	(45.1)	433.7^+^	(60.5)	614.0^+^	(NA)
Ectopic inner foveal layer thickness (um), mean (SD)	7.5	(22.9)	12.1	(26.0)	27.4	(38.0)	161.3^+^	(40.1)	362.0^+^	(NA)
Outer nuclear layer thickness (um), mean (SD)	125.9	(36.0)	142.9	(36.9)	200.6^+^	(41.9)	205.7^+^	(13.9)	177.0^+^	(NA)
Ellipsoid zone disruption, n (% of type)	3	(6.0%)	4	(7.1%)	0	(0.0%)	0	(0.0%)	1	(100.0%)
Intraretinal cysts, n (% of type)	6	(12.0%)	14	(25.0%)	6	(66.7%)	2	(66.7%)	1	(100.0%)
Subretinal fluid, n (% of type)	0	(0.0%)	2	(3.6%)	1	(11.1%)	0	(0.0%)	0	(0.0%)
Mean visual acuity, LogMAR (SD)	0.3	(0.4)	0.2	(0.3)	0.2	(0.2)	0.9	(0.9)	0.6	(NA)
Mean snellen visual acuity	20/40		20/30		20/30		20/160*		20/80*	

^*^One way ANOVA with Bonferroni post hoc analysis showed that visual acuity for Stage 3 ERMs was significantly lower than stage 0 (p = 0.04), stage 1 (p = 0.02), and stage 2 ERMs (p = 0.04)

^+^p < 0.05, One way ANOVA with Bonferroni post hoc analysis (vs stage 0)

**Fig. 2:**
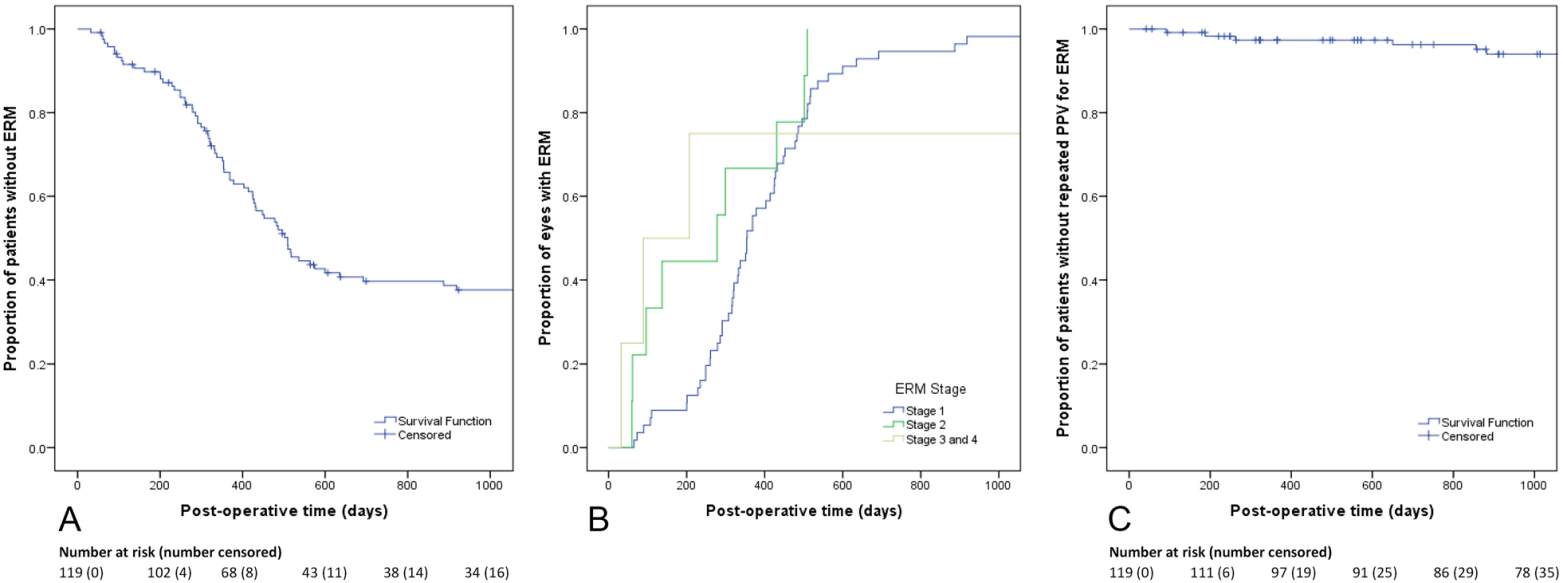
Survival analysis of ERM formation and surgery after PPV for RRD. **A** Kaplan–Meier survival curve for time ERM formation. **B** Inverse Kaplan–Meier survival curve for time to ERM formation by stage of ERM. **C** Kaplan–Meier survival curve for time to secondary ERM surgery

Survival analysis of ERM formation is shown in Fig. 2A. Kaplan–Meier survival analysis revealed that 39.7% of eyes at 2 years did not form ERMs (Fig. 2A). Only half of ERMs formed within the first post-operative year (n = 35/69, 51%). Time to ERM formation by ERM stage is shown in Fig. 2B. Median estimates of time to ERM formation were 354 days for stage 1, 278 days for stage 2, and 89 days for stage 3/4 ERMs (p < 0.05). Survival analysis of ERM surgery is shown in Fig. 2C. Kaplan–Meier survival analysis revealed that 96.3% of eyes at 2 years did not require ERM surgery. Only half of ERMs were peeled within the first post-operative year (n = 3/6, 50%).and the mean time to reoperation was 488 ± 351 days. Eyes that underwent subsequent ERM peeling did so for stage 3 ERM or worse (n = 3, 50%), decreasing VA with ERM (n = 2, 33%), and dislocated IOL with ERM (n = 1, 17%) (Table 4).

**Table 4:** Preoperative and intraoperative characteristics of patients requiring postoperative epiretinal membrane peeling

Patient	ERM stage	Highest BCVA until ERM detection (LogMAR, Snellen)	Pre ERM Peel BCVA (LogMAR, Snellen)	Time to ERM surgery (days)	Macula status of initial RD	Initial detachment size (clock hours)	Number of tears during initial RD	Intra-operative cryotherapy	360 laser	Failed pneumatic	Post ERM peel VA (LogMAR, Snellen)	Time post peel	Indication for ERM peeling
1	3	0.7 (= 20/100)	0.9 (= 20/150)	188	OFF	5	4	Yes	Yes	Yes	0.2 (= 20/30)	2 years	Visually significant ERM
2	1	0.5 (= 20/60)	0.6 (= 20/80)	856	OFF	6	3	No	No	No	0.6 (= 20/80)	1 year	Dislocated IOL with ERM causing focal Extrafoveal traction
3	2	0.2 (= 20/30)	0.6 (= 20/80)	650	OFF	5	6	No	Yes	No	0.0 (= 20/20)	2 years	Decrease in VA due to progressive ERM
4	4	0.5 (= 20/70)	0.6 (= 20/80)	262	ON	12	4	No	Yes	Yes	0.2 (= 20/30)	2 years	Severe visually significant ERM
5	1	0.3 (= 20/40)	0.5 (= 20/60)	882	OFF	7	1	No	Yes	Yes	0.5 (= 20/60)	1 year	Symptomatic ERM due to focal Extrafoveal traction
6	3	1.9 (= 20/1450)	1.9 (= 20/1450)	91	OFF	4	4	No	Yes	No	0.2 (= 20/30)	2 years	Visually significant ERM with cataract

*ERM* epiretinal membrane, *BCVA* best corrected snellen visual acuity, *RD* retinal detachment, *PVR* proliferative vitreoretinopathy, *IOL* intraocular lens

Risk factors for ERM formation included intraoperative cryotherapy (HR = 2.5, CI 1.3–4.9, p < 0.01), ≥ 1000 laser shots (HR = 2.2, CI 1.3–3.6, p < 0.01), choroidal detachment (HR = 7.2, CI 1.7–30.7, p < 0.01) and 360° retinal photocoagulation (HR = 2.0, CI 1.1–3.5, p < 0.02). There was a trend for 3 or more tears (HR = 6.3, CI 0.7–56.0, p = 0.1), and ≥ 1000 laser shots (HR = 4.6, CI 0.5–44.8, p = 0.18) to be risk factors for ERM surgery. The univariate cox proportional hazards model failed to identify other preoperative or intraoperative risk factors for ERM formation or surgery, including prior pneumatic retinopexy, extent of detachment, macular status (on or off), time to surgery and duration of surgery (p > 0.05, Table 5).

**Table 5:** Preoperative risk factors for post-operative epiretinal membrane formation and membrane peeling

	Interval	ERM formation				ERM surgery			
		Hazard ratio	95% CI		P value	Hazard ratio	95% CI		P value
			Lower	Upper			Lower	Upper	
Female	vs male	0.9	0.5	1.5	0.68	2.3	0.5	11.4	0.31
Left Eye	vs right eye	0.9	0.6	1.5	0.75	0.9	0.2	4.6	0.92
Failed pneumatic	vs no failed pneumatic	1.0	0.6	1.6	0.89	2.0	0.4	10.1	0.38
Retinal detachment greater than 50%	vs 50% or less	1.5	0.9	2.4	0.14	1.5	0.3	9.2	0.64
PVR Grade A or B	vs no PVR	1.2	0.3	5.0	0.77	0.0	0.0	4.1e [9]	0.81
Choroidal detachment*	Vs no choroidal detachment	7.2	1.7	30.7	0.01	0.0	0.0	3.7e [11]	0.84
Cryotherapy*	vs no cryotherapy	2.5	1.3	4.9	0.01	1.8	0.2	15.5	0.59
Laser shots ≥ 1000*	vs laser shots < 1000	2.2	1.3	3.6	0.01	4.6	0.5	44.8	0.18
3 or more tears	vs 2 tears or less	1.2	0.8	2.0	0.37	6.3	0.7	56.0	0.10
Combined cataract surgery	vs no combined cataract surgery	1.1	0.6	2.0	0.77	2.3	0.4	12.4	0.35
Vitreous hemorrhage	vs no vitreous hemorrhage	0.8	0.4	1.9	0.63	0.0	0.0	3541.8	0.58
360° photocoagulation*	vs no 360° photocoagulation	2.0	1.1	3.5	0.02	2.2	0.3	18.9	0.47
Macula off	vs macula on	1.1	0.7	1.8	0.67	3.2	0.4	27.5	0.29

*PVR* proliferative vitreoretinopathy, *Q* quartile

*p < 0.05

^+^p = 0.10

Mean visual acuity at the time of diagnosis of ERM was significantly better in eyes that were observed vs eyes that underwent ERM peeling (0.23 ± 0.33 LogMAR (Snellen = 20/34) vs 0.82 ± 0.53 (20/130), p < 0.05, Table 6). Eyes that were observed without peeling maintained their visual acuity, with a mild improvement noted at last followup (0.23 ± 0.33 LogMAR (20/34) vs 0.18 ± 0.53 (20/30), p < 0.05, last followup 28 ± 17 months after diagnosis of ERM). Half of patients (n = 3/6) who underwent peeling had a 0.2 LogMAR worsening from highest postoperative BCVA until ERM detection, with 2 others experiencing a 0.1 LogMAR worsening (Table 4). All patients who underwent ERM peeling had worse than 20/60 VA pre peeling, with one having 20/1450 VA due to combined cataract and ERM. Patients who underwent ERM peeling had an improvement in visual acuity at last followup compared to pre-peeling which trended toward statistical significance (0.82 ± 0.53 pre (20/130) vs 0.39 ± 0.20 post (20/50, p = 0.16, last followup 18 ± 18 months after peeling, Table 6). For all patients, mean visual acuity improved at 24 months compared to pre-operatively (0.89 ± 0.87 LogMAR (20/155) vs 0.29 ± 0.54 (20/40), p < 0.001).

**Table 6:** Visual acuity in eyes with epiretinal membrane that were observed vs underwent epiretinal membrane peeling

	ERM stage	VA at diagnosis of ERM (LogMAR, Snellen)	SD	VA at last followup (LogMAR, Snellen)	SD	Time to last followup (months)	SD
ERM without peeling	1	0.24 (= 20/34)	0.34	0.19 (= 20/31)	0.36	27.6	16.5
	2	0.20 (= 20/32)	0.21	0.07 (= 20/24)	0.11	31.0	22.1
	3	0.18 (= 20/30)	–	0.18 (= 20/30)	–	18.8	–
	4	–	–	–	–	–	–
	All	0.23 (= 20/34)*^+^	0.33	0.18 (= 20/30)^+^	0.34	27.9	17.1
ERM with peeling	1	0.48 (= 20/60)	0.00	0.54 (= 20/70)	0.09	42.0	8.5
	2	0.60 (= 20/80)	–	0.60 (= 20/80)	-	12.0	–
	3	1.37 (= 20/400)	0.70	0.24 (= 20/34)	0.09	36.0	17.0
	4	0.60 (= 20/80)	–	0.18 (= 20/30)	–	36.0	–
	All	0.82 (= 20/132)*	0.53	0.39 (= 20/50)	0.20	18.0	18.3

*VA* visual acuity, *ERM* epiretinal membrane, *SD* standard deviation

*p < 0.05 Visual acuity was significantly worse at diagnosis of ERM in eyes that later underwent ERM peeling compared to eyes that were observed

^+^p < 0.05 There was a significant improvement in visual acuity in eyes with ERM that were observed without peeling

### Failure to cure and complications

Incidences of postoperative ERM formation, re-detachment and complications are summarized in Table 2. ERM formation occurred in approximately half of patients (n = 69, 58%). However, few of these ERMs required surgical removal (n = 6, 5%). Re-detachment occurred in 2 eyes in the late post-operative period (2%). Other complications included the formation of grade C proliferative vitreoretinopathy (n = 2, 2%), macular holes (n = 4, 3%), severe macular edema (n = 1, 0.8%) and subretinal perfluorocarbon (n = 1, 0.8%). Of the eyes that developed macular holes, 2 (2%) were full thickness which required subsequent vitrectomy with ILM peeling.

## Discussion

While several studies have evaluated risk factors for ERM formation after uncomplicated PPV for primary RRD, this is one of the first studies to directly assess preoperative and intraoperative risk factors for ERM formation and ERM peeling in patients with clinically significant visual symptoms and OCT correlation of tractional structural macular changes.

ERM formation after PPV for primary RRD was very common in our cohort, with more than half of eyes (n = 69/119, 58%) developing an ERM visible on OCT. Fortunately, the majority were Stage 1 or 2 ERMs (n = 65, 55%) with no impact on visual acuity. Only 5% (n = 6) of eyes (9% of ERMs (6/69)) required subsequent PPV for ERM peeling and half of peelings (50%) took place within the first post-operative year.

Our findings are consistent with ERM formation rates in the recent literature [4, 6–8]. The formation of ERMs that are severe enough to warrant surgical intervention also vary in the literature, with rates as low as 1.6% up to 35% of eyes after PPV for primary RRD [16]. A recent meta-analysis of 586 eyes showed that an average of 16% of eyes required a second vitrectomy for ERM peeling [10]. The rate of re-operation was lower in our cohort, possibly due to advancements in surgical tools and techniques (including smaller gauge vitrectomy (25-gauge vs 20-gauge) and less use of cryotherapy). This decreased reoperation rate is despite a longer follow-up in our cohort (30 months vs 6–16 months) [3, 16–19]. This re-enforces that visually significant ERM formation is most likely to occur early in the post-operative course. However, it is possible to have symptomatic ERMs that present long term, with 3 reoperations for ERM peeling (50%) occurring after the first post-operative year in our cohort.

The formation of ERM and need for subsequent surgery is extremely low/almost zero following prophylactic ILM peeling during PPV for primary RRD [10]. Studies directly comparing prophylactic ILM peeling vs PPV alone have shown an average absolute risk reduction of subsequent ERM surgery of 16%, with a number needed to treat (NNT) of 6.25. In our cohort, the rate of ERM peeling was almost a quarter of that seen in the literature (5%), resulting in a NNT of 20. If we included the two cases of full thickness macular holes which may have been prevented by prophylactic ILM peeling, this decreased the NNT to 15. Prophylactic ILM peeling is also not without risk, with potential complications including retinal hemorrhage, eccentric scotoma, retinal edema, vitreous hemorrhage and photopic and stain related toxicity [20–23]. Given the low rate of subsequent surgery, the uncertain potential gain in final VA and the added risk of prophylactic ILM peeling during the repair of primary RRD, our data suggests that the addition of prophylactic peeling warrants caution.

In our cohort, we found several risk factors for epiretinal membrane formation and possible risk factors for ERM surgery. Intraoperative cryotherapy and greater than 1000 laser shots both doubled the risk of ERM formation. Choroidal detachment increased this risk by a factor of 7. Other retrospective cohort studies have shown increased rates of ERM formation following PPV for equatorial vs anterior breaks [18], multiple retinal breaks [8], and retinal breaks greater than 2 disc diameters [8]. One study did not see an increased rate of ERM formation in eyes with macula off detachments [18]. One possibility for this difference is that that these studies all examined the risk of formation of ERM, not the risk for the need of ERM peeling/secondary surgery. The only factors that trended towards significance in our cohort were 3 or more tears present in the initial detachment (HR = 6.3, p = 0.1) and more than 1000 laser shots (HR = 4.6, p = 0.18). Surgeons will consider multiple factors when it comes to ERM peeling post primary PPV, including structural OCT changes but most importantly visual symptoms. Patients may also be bothered by significant metamorphopsia due to the ERM. Unfortunately, the degree of metamorphopsia was not measured in this retrospective study. Interestingly, prior failed pneumatic retinopexy and extent of retinal detachment were not associated with increased ERM surgery in our cohort. Unlike other reports looking at prophylactic 360° laser for primary RRD that have failed to demonstrate increased risk for ERM formation [24], our data does show a twofold increase in risk of ERM formation using this technique.

Cryotherapy is thought to induce proliferative vitreoretinopathy by triggering the dispersion of retinal pigment epithelial cells (RPE) cells through a retinal break [25]. However, there are few studies directly comparing the formation of ERMs or other PVR with cryotherapy vs laser retinopexy. A recent large study evaluating eyes with retinal breaks with no detachment showed no difference in ERM progression resulting in surgical intervention between eyes treated with cryotherapy versus laser retinopexy [26]. In our study, both large amounts of laser and the use of cryotherapy doubled the risk of ERM formation. This is possibly due to greater dispersion of RPE cells and a larger inflammatory response following surgery for RRD.

There is no current gold standard treatment of RRD due to a lack of large randomized clinical trials showing consistent differences among other highly effective treatment options including pneumatic retinopexy (PR) and scleral buckling (SB) [27]. Knowledge on the frequency and severity of sequelae and complications, such as ERM formation associated to PPV, PR and SB may help guide surgical decision making [28]. Previous reports on SB have identified macula-off detachments, pre-operative PVR and previous surgery as risk factors for ERM formation [29, 30]. A study directly comparing the complications of these techniques would greatly help in decision making.

As this was a retrospective study, there was no predetermined criteria for when to perform ERM peeling in our patients. However, upon analysis of the data it was clear that visual acuity, and visual potential, was a main criterion that influenced our surgeons. Average Snellen visual acuity in patients with ERM that were observed without peeling was 20/30 and did not change over many months of followup. There were no patients with visual acuity worse than 20/50 that were observed, excluding patients with pre-existing low vision from corneal scarring (n = 1) or glaucoma (n = 2). Patients that underwent ERM peeling had poorer visual acuity at diagnosis of ERM, averaging 20/130 snellen visual acuity, and demonstrated worsening of visual acuity (0.2 logMAR in 3/6 eyes, 0.1 LogMAR in 2/6 eyes). This pre-peeling preoperative visual acuity was similar to that seen in a previous report by Rao et al. [31] Other studies have noted similar visual acuity thresholds for ERM peeling post RD, including worse than 20/40 visual acuity or metamorphopsia (vs 20/50 in our group) [19], and a decrease in visual acuity by 0.2 logMAR or more at ERM detection. [32]

### Limitations

This retrospective cohort study was limited by some loss to follow-up (n = 103/119; 86% eyes retained at 3 years). To improve the quality of the data, patients were excluded if they had less than 6 months of follow-up (n = 8). Follow-up in the first year of the study was excellent, where the majority of ERM formation is known to occur [n = 115/119 (96%)]. There were few patients that required ERM surgery, which made Cox regression modelling underpowered. A larger sample size would be needed to further validate these results. There were also no pre-established criteria between surgeons on threshold for reoperation of ERMs. Lack of preoperative OCT for all patients was also a limitation for this study and may have led to overcalling ERM formation in the early post-operative period in some eyes. However, the large majority of ERMs were identified after 6 months postoperatively (n = 57/69, 83%), after a post-operative OCT not showing an ERM, so this overestimation would have minimal impact on the interpretation of the results. Any patients with ERM visible on preoperative clinical exam were excluded. It is also possible that ERM detection may have been improved with en-face OCT imaging, which was not part of the OCT based ERM classification system used in this study. The strengths of this study include confirmation of ERM by OCT with a modern grading system and visual acuity pairing, as well almost twice the average follow-up time compared to previous cohorts in the literature (typically 12–16 months).

## Conclusion

In summary, we found that over half of eyes undergoing RRD repair using PPV developed ERMs, but only 5.0% of eyes required subsequent surgery for ERM removal. Our results suggest that pneumatic retinopexy was not a risk factor for significant ERM formation, but risk of formation was elevated with intraoperative use of cryopexy and 360 prophylactic laser. Due to the low rate of secondary surgery, prophylactic ILM peeling during primary uncomplicated PPV for RRD is not performed at our institution. These findings need to be further investigated in a larger controlled, prospective trial before a more general recommendation can be offered.

## Data Availability

Available upon request.

## References

[1] Schwartz SG, Flynn HW, Mieler WF (2013). Update on retinal detachment surgery. Curr Opin Ophthalmol.

[2] Fallico M (2018). Internal limiting membrane peeling versus no peeling during primary vitrectomy for rhegmatogenous retinal detachment: a systematic review and meta-analysis. PLoS ONE.

[3] Forlini M (2018). Comparative analysis of retinal reattachment surgery with or without internal limiting membrane peeling to prevent postoperative macular pucker. Retina.

[4] Katira RC (2008). Incidence and characteristics of macular pucker formation after primary retinal detachment repair by pars plana vitrectomy alone. Retina.

[5] Antaki F (2020). Predictive modeling of proliferative vitreoretinopathy using automated machine learning by ophthalmologists without coding experience. Sci Rep.

[6] Council MD (2005). Visual outcomes and complications of epiretinal membrane removal secondary to rhegmatogenous retinal detachment. Ophthalmology.

[7] Banker TP (2015). Epiretinal membrane and cystoid macular edema after retinal detachment repair with small-gauge pars plana vitrectomy. Eur J Ophthalmol.

[8] Matoba R (2021). Assessment of epiretinal membrane formation using en face optical coherence tomography after rhegmatogenous retinal detachment repair. Graefe’s Arch Clin Exp Ophthalmol.

[9] Lee GW (2020). Characteristics of secondary epiretinal membrane due to peripheral break. Sci Rep.

[10] Yannuzzi NA (2018). Internal limiting membrane peeling during pars plana vitrectomy for rhegmatogenous retinal detachment: cost analysis, review of the literature, and meta-analysis. Retina.

[11] Azuma K (2017). Effects of internal limiting membrane peeling combined with removal of idiopathic epiretinal membrane: a systematic review of literature and meta-analysis. Retina.

[12] Govetto A (2017). Insights into epiretinal membranes: presence of ectopic inner foveal layers and a new optical coherence tomography staging scheme. Am J Ophthalmol.

[13] Machemer R (1991). An updated classification of retinal detachment with proliferative vitreoretinopathy. Am J Ophthalmol.

[14] Holladay JT (1997). Proper method for calculating average visual acuity.

[15] Schulze-Bonsel K (2006). Visual acuities “hand motion” and “counting fingers” can be quantified with the freiburg visual acuity test. Invest Ophthalmol Vis Sci.

[16] Obata S (2021). Effect of internal limiting membrane peeling on postoperative visual acuity in macula-off rhegmatogenous retinal detachment. PLoS ONE.

[17] Garweg JG (2019). Impact of inner limiting membrane peeling on visual recovery after vitrectomy for primary rhegmatogenous retinal detachment involving the fovea. Retina.

[18] Martínez-Castillo V (2012). Epiretinal membrane after pars plana vitrectomy for primary pseudophakic or aphakic rhegmatogenous retinal detachment incidence and outcomes. Retina.

[19] Nam KY, Kim JY (2015). Effect of internal limiting membrane peeling on the development of epiretinal membrane after pars plana vitrectomy for primary rhegmatogenous retinal detachment. Retina.

[20] Kwok A (2002). Vision threatening vitreous haemorrhage after internal limiting membrane peeling in macular surgeries. Br J Ophthalmol.

[21] Karacorlu M, Karacorlu S, Ozdemir H (2003). Iatrogenic punctate chorioretinopathy after internal limiting membrane peeling. Am J Ophthalmol.

[22] Haritoglou C (2001). Macular changes after peeling of the internal limiting membrane in macular hole surgery. Am J Ophthalmol.

[23] Gandorfer A, Haritoglou C, Kampik A (2008). Toxicity of indocyanine green in vitreoretinal surgery. Dev Ophthalmol.

[24] Dirani A (2020). 360-degree intra-operative laser retinopexy for the prevention of retinal re-detachment in patients treated with primary pars plana vitrectomy. Graefes Arch Clin Exp Ophthalmol.

[25] Campochiaro PA (1985). Cryotherapy enhances intravitreal dispersion of viable retinal pigment epithelial cells. Arch Ophthalmol.

[26] Blackorby BL (2019). Epiretinal membrane formation after treatment of retinal breaks: cryoretinopexy versus laser retinopexy. Ophthalmology retina.

[27] Velez-Montoya R (2016). Primary repair of moderate severity rhegmatogenous retinal detachment: a critical decision-making algorithm. Med Hypothesis Discov Innov Ophthalmol.

[28] Xu ZY (2021). Reporting of complications in retinal detachment surgical trials: a systematic review using the CONSORT extension for harms. JAMA Ophthalmol.

[29] Tanenbaum HL (1970). Macular pucker following retinal detachment surgery. Arch Ophthalmol.

[30] Lobes LA, Burton TC (1978). The incidence of macular pucker after retinal detachment surgery. Am J Ophthalmol.

[31] Rao RC, Blinder KJ, Smith BT, Shah GK (2013). Internal limiting membrane peeling for primary rhegmatogenous retinal detachment repair. Ophthalmology.

[32] Akiyama K, Fujinami K, Watanabe K, Tsunoda K, Noda T (2016). Internal limiting membrane peeling to prevent post-vitrectomy epiretinal membrane development in retinal detachment. Am J Ophthalmol.

